# Candidate master microRNA regulator of arsenic-induced pancreatic beta cell impairment revealed by multi-omics analysis

**DOI:** 10.1007/s00204-022-03263-9

**Published:** 2022-03-21

**Authors:** Jenna E. Todero, Kieran Koch-Laskowski, Qing Shi, Matt Kanke, Yu-Han Hung, Rowan Beck, Miroslav Styblo, Praveen Sethupathy

**Affiliations:** 1 Department of Biomedical Sciences, College of Veterinary Medicine, Cornell University, Ithaca, NY USA; 2 Department of Nutrition, Gillings School of Public Health, University of North Carolina at Chapel Hill, Chapel Hill, NC USA

**Keywords:** Arsenic, Beta cells, MicroRNAs, Diabetes, Sequencing, Insulin secretion

## Abstract

Arsenic is a pervasive environmental toxin that is listed as the top priority for investigation by the Agency for Toxic Substance and Disease Registry. While chronic exposure to arsenic is associated with type 2 diabetes (T2D), the underlying mechanisms are largely unknown. We have recently demonstrated that arsenic treatment of INS-1 832/13 pancreatic beta cells impairs glucose-stimulated insulin secretion (GSIS), a T2D hallmark. We have also shown that arsenic alters the microRNA profile of beta cells. MicroRNAs have a well-established post-transcriptional regulatory role in both normal beta cell function and T2D pathogenesis. We hypothesized that there are microRNA master regulators that shape beta cell gene expression in pathways pertinent to GSIS after exposure to arsenicals. To test this hypothesis, we first treated INS-1 832/13 beta cells with either inorganic arsenic (iAs^III^) or monomethylarsenite (MAs^III^) and confirmed GSIS impairment. We then performed multi-omic analysis using chromatin run-on sequencing, RNA-sequencing, and small RNA-sequencing to define profiles of transcription, gene expression, and microRNAs, respectively. Integrating across these data sets, we first showed that genes downregulated by iAs^III^ treatment are enriched in insulin secretion and T2D pathways, whereas genes downregulated by MAs^III^ treatment are enriched in cell cycle and critical beta cell maintenance factors. We also defined the genes that are subject primarily to post-transcriptional control in response to arsenicals and demonstrated that miR-29a is the top candidate master regulator of these genes. Our results highlight the importance of microRNAs in arsenical-induced beta cell dysfunction and reveal both shared and unique mechanisms between iAs^III^ and MAs^III^.

**Supplementary Information:**

The online version contains supplementary material available at 10.1007/s00204-022-03263-9.

## Introduction

Inorganic arsenic (iAs) is a potent and ubiquitous environmental toxin that is ranked as the number one priority for investigation by the Agency for Toxic Substances and Disease Registry (ASTDR) (Stýblo et al. 2021; Chung et al. 2014; ASTDR 2020). Chronic exposure to iAs has been associated with numerous medical complications, such as cancer and cardiovascular disease (Sarkar and Paul 2016), and acts through a variety of different mechanisms (Khairul et al. 2017; Hughes 2002; Nurchi et al. 2020). iAs and its methylated trivalent metabolites, monomethylarsenite (MAs^III^) and dimethylarsenite (DMAs^III^), are known to exert toxic effects in a tissue-specific and arsenical-specific manner (Stýblo et al. 2021; Styblo et al. 2000). Arsenicals are established human diabetogens, though the underlying mechanisms remain unclear (Maull et al. 2012; Navas-Acien Ana et al. 2006).

Type 2 diabetes (T2D) is a multi-faceted disease, with both genetic and environmental risk factors (Kahn 2003, 2; Langenberg and Lotta 2018; Huang and Hu 2015), which is marked by impaired beta cell function; specifically, defective glucose-stimulated insulin secretion (GSIS) (American Diabetes Association 2004; Christensen and Gannon 2019; Al-Sulaiti et al. 2019). T2D affects over 400 million people worldwide and it is estimated that 10% of global health expenses are spent on diabetes treatment (IDF Atlas 9th Edition). Given this costly toll, it is imperative that we identify mechanisms of disease onset and progression toward the goal of novel and effective therapeutic interventions. Although iAs exposure is associated with diabetes-related phenotypes, the underlying molecular mechanisms of disease pathogenesis remain largely unknown.

Previous studies by our group and others have shown that trivalent arsenicals impair GSIS in both isolated murine islets as well as rodent beta cell lines (Douillet et al. 2013; Huang et al. 2019; Díaz-Villaseñor et al. 2006). More recently, we have demonstrated that iAs^III^ alters the microRNA (miRNA) landscape in beta cells much more substantially than other metals that can also affect GSIS, suggesting that miRNAs may play an important role in the mechanisms underlying the inhibition of GSIS by iAs^III^ (Beck et al. 2019; Beck et al. 2017).

MiRNAs are short non-coding RNA molecules that regulate gene expression post-transcriptionally (Bartel 2004). Studies over the past 15 + years have established roles for miRNAs in beta cell survival, proliferation, and function, including insulin secretion (Poy et al. 2004; Poy et al. 2009; Bagge et al. 2012; Belgardt et al. 2015; Eliasson and Esguerra 2014; Vienberg et al. 2017). Though we have shown that iAs^III^ alters miRNA expression in INS-1 832/13 beta cells, it has not been reported whether MAs^III^, a potent inhibitor of GSIS (Douillet et al. 2013), has a similar effect. Also, it is completely unknown which, if any, miRNAs are the primary drivers of altered gene expression in beta cells after exposure to arsenicals. To address this knowledge gap, we implemented a multi-omics analysis pipeline that integrates information from state-of-the-art chromatin run-on sequencing (ChRO-seq), RNA-sequencing (RNA-seq), and small RNA-sequencing (smRNA-seq). This novel integrative genomics approach identified a candidate master miRNA regulator of the effects of arsenicals on beta cells. The results of this study highlight the importance of miRNAs in arsenical-induced beta cell dysfunction and reveal both shared and unique mechanisms between iAs^III^ and MAs^III^.

## Methods

### Cell culture

The INS-1 832/13 rat insulinoma beta cell line was maintained at 5% CO_2_, 37 °C in RPMI 1640 media (Gibco, Waltham, MA, USA) supplemented with 2 mM L-glutamine media (Gibco, Waltham, MA, USA), 10% FBS, 10 mM HEPES media (Gibco, Waltham, MA, USA), 1 mM sodium pyruvate media (Gibco, Waltham, MA, USA), 100 U/mL penicillin media (Gibco, Waltham, MA, USA), 100 μg/mL streptomycin media (Gibco, Waltham, MA, USA), and 0.05 mM β-mercaptoethanol (Sigma, St. Louis, MO, USA). INS-1 832/13 cells were exposed to iAs^III^ (iAs^III^; sodium arsenite > 99% pure; Sigma-Aldrich, St. Louis, MO, USA) or MAs^III^ (methylarsine oxide, > 98% pure) for 24 h prior to GSIS and chromatin or RNA isolation.

### Glucose stimulated insulin secretion assay

As previously described (Beck et al. 2019), INS-1 832/13 cells were seeded at 1,000,000 cells/well in a 12-well plate 24 h prior to beginning experiments. Cells were then exposed to either iAs^III^ or MAs^III^ for 24 h. After 24 h cells cell culture media was replaced with secretion assay buffer (SAB), which consists of 114 mM NaCl, 4.7 MM KCl, 1.2 mM KH_2_PO_4_, 1.16 mM MgSO_4_, 20 mM HEPES, 2.5 mM CaCl_2_, 0.2% bovine serum albumin, 25.5 mM NaHCO_3_, and 0 mM of glucose. Cells remained in glucose-free media for 40 min before incubating in SAB with 2.5 mM of glucose for 60 min followed by an SAB with 16.7 mM glucose incubation for 2 h. Aliquots of media were collected at 2.5 mM and 16.7 mM glucose incubations. Insulin levels were detected using the Ultra Sensitive Mouse Insulin ELISA Kit (Crystal Chem) and normalized to cellular protein. Biological replicates are shown as the mean with the ± standard error. A two-tailed unpaired Student’s *t* test was used for statistical analysis using a *p* value threshold < 0.05.

### RNA isolation and sequencing

Total RNA was isolated using the Total RNA Purification Kit (Norgen Biotek, Thorold, Ontario, Canada) and quantified using the Nanodrop 2000 (Thermo Fisher Scientific, Waltham, MA). RNA integrity was assessed using the 4200 Tapestation (Agilent Technologies, Santa Clara, CA). Isolated RNA was used to make libraries for both smRNA-sequencing and RNA-sequencing. SmRNA-sequencing libraries were prepared by the Genome Sequencing Facility of Greehey Children’s Cancer Research Institute at the University of Texas Health Science Center at San Antonio using the TriLink CleanTag Small RNA Ligation kit (TriLink Biotechnologies, San Diego, CA). Eight libraries were sequenced per lane with single-end 50 × on the HiSeq3000 platform. RNA-sequencing libraries (polyA +) were prepared at Cornell’s Transcriptional Regulation and Expression Facility (TREx) using NEB Next Ultra II kits (New England BioLabs, Ipswich MA). Paired-end sequencing was performed at 20 million reads/sample on the NextSeq500 platform (Illumina, San Diego, CA).

### ChRO-seq library preparation and sequencing

Chromatin isolation was performed as previously described (Mahat et al. 2016; Chu et al*.* 2018). Control and arsenical-treated INS-1 832/13 cells were pelleted and then resuspended in cold 1X NUN lysis buffer [0.3 M NaCl, 1 M Urea, 1% (v/v) NP-40, 20 mM HEPES pH 7.5, 7.5 mM MgCl_2_, 0.2 mM EDTA, 1 mM DTT, 50U/mL SUPERase In RNAse Inhibitor (Thermo Fisher Scientific, Waltham, MA), and 1× Protease Inhibitor Cocktail (Roche, Basel, Switzerland)]. Samples were incubated at 12 °C in a Thermomixer (Eppendorf, Hamburg, Germany) at 2000 rpm for 30 min and then centrifuged at 12,500 ×*g* for 30 min at 4 °C. Resultant chromatin pellets were washed three times with 1 mL wash buffer [50 mM Tris–HCl pH 7.5 supplemented with 40U/mL RNase Inhibitor], and centrifuged at 10,000 ×*g* for 5 min at 4 °C between washes. After the final wash step, samples were stored in chromatin storage buffer [50 mM Tris–HCl pH 8.0, 25% (v/v) glycerol, 5 mM magnesium acetate, 0.1 mM EDTA, 5 mM DTT, and 40U/mL RNase Inhibitor], loaded into a Biorupter at 4 °C (Diagenode, Denville, NJ), and sonicated to solubilize the chromatin pellets into solution.

Using solubilized chromatin, run-on reactions were performed by mixing each sample with an equal volume of 2× run-on reaction mix [10 mM Tris–HCl pH 8.0, 5 mM MgCl_2_, 1 mM DTT, 300 mM KCl, 400 µM ATP, 0.8 µM CTP, 400 µM GTP, 400 µM UTP (NEB, Ipswich, MA), 40 µM Biotin-11-CTP (Perkin Elmer, Waltham, MA), 100 ng yeast tRNA (VWR, Radnor, PA), 0.8U/µL RNase Inhibitor, and 1% (w/v) sarkosyl]. The samples were then incubated in a Thermomixer at 750 rpm for 5 min at 37 °C. Run-on reactions were terminated by adding TRIzol LS (Thermo Fisher Scientific, Waltham, MA) and incubating the samples at room temperature. Following phenol–chloroform extraction, samples were pelleted with GlycoBlue (Thermo Fisher Scientific, Waltham, MA) to visualize the nascent RNA. Pellets were then resuspended in DEPC-treated water and heat denatured at 65 °C for 40 s. To fragment nascent RNA molecules, base hydrolysis was performed by incubating samples with 0.2 N NaOH on ice for 4 min followed by the addition of 1 M Tris–HCl pH 6.8 and subsequent cleanup via Micro Bio-Spin P-30 gel columns (Bio-Rad, Hercules, CA).

These nascent RNA samples were used to construct ChRO-seq libraries through the following procedures: (i) 3′ adapter ligation with T4 RNA Ligase 1 (NEB, Ipswich, MA); (ii) Streptavidin bead binding; (iii) 5′ de-capping with RNA 5′ pyrophosphohydrolase (NEB, Ipswich, MA); (iv) 5′ end phosphorylation using T4 polynucleotide kinase (NEB, Ipswich, MA) followed by TRIzol extraction; (v) 5′ adapter ligation with T4 RNA Ligase 1, in which the adapter contained a 6-nucleotide unique molecular identifier (UMI) for bioinformatic detection and elimination of PCR duplicates in downstream data processing steps; (vi) Streptavidin bead binding followed by TRIzol extraction; (vii) cDNA synthesis by reverse transcription via SuperScript IV Reverse Transcriptase (Thermo Fisher Scientific, Waltham, MA); and (viii) PCR library amplification with the Q5 High-Fidelity DNA Polymerase (NEB, Ipswich, MA). The resultant libraries were sequenced (5′ single end; single-end 75×) using the NextSeq500 high-throughput sequencing system (Illumina, San Diego, CA) at the Cornell University Biotechnology Resource Center Genomics Facility.

### Bioinformatics analysis

Small RNA-seq reads were processed using miRquant 2.0 (Kanke et al. 2016). In brief, the 3′ sequencing adapter was removed from the read, reads larger than 14nt were aligned to the rat genome (rn6), and aligned reads were quantified. Any reads aligning to miRNAs loci were annotated according to miRbase (v18). Differential miRNA expression was determined using DESeq2 (Love et al. 2014). RNA sequencing reads were aligned to the rat genome (rn6) using STAR (v2.4.2a) (Dobin et al. 2013), and reads aligning to the transcriptome were quantified using Salmon (Patro et al. 2017). Differential gene expression across treatment groups was determined using DESeq2 (Love et al. 2014). The design used was ~ batch + condition, where condition is the arsenical used (except in the analysis of INS-1 832/13 cells exposed to MAs^III^, for which the design used was ~ condition).

ChRO-seq data were analyzed using an established bioinformatic pipeline (Chu et al*.* 2019). First, PCR duplicates were removed by UMI collapsing and trimming with PRINSEQ lite 0.20.2 (Schmieder and Edwards 2011). 3′ adapters were trimmed from the remaining reads using Cutadapt 1.16 (Martin 2011) with a maximum 10% error rate. Next, reads were mapped using Burrows-Wheeler Aligner to the annotated rn6. The location of active RNA polymerase was represented by a single base that denotes the 3′ end of the nascent RNA, which corresponds to the position on the 5′ end of each sequenced read. Analysis of differentially transcribed (DT) genes was performed using stranded ChRO-seq signals present in annotated gene bodies, excluding reads within 500 bases downstream of the transcription start site to avoid bias due to the pausing of RNA polymerase at promoters. Furthermore, gene bodies of less than 1000 bases were excluded given the bias introduced against shorter genes with the removal of the aforementioned pause peak. DT genes across treatment groups were identified and filtered by DESeq2 (Love et al. 2014) analysis using two stages per comparison (i.e., iAs^III^ vs. Control or MAs^III^ vs. Control).

Post-transcriptionally regulated genes were identified first by applying DESeq2 two-factor analysis in order to define post-transcriptionally unstable or stable genes (adjusted p value < 0.2). Then we filtered for genes which were unchanged at the transcriptional level (i.e., baseMean > 100 across all samples and an adjusted p value > 0.2 by ChRO-seq analysis), but changed at the steady-state gene expression level (i.e., baseMean > 100 across all samples, log2fold-change > 0.5 or < −0.5, and an adjusted p value < 0.2 by RNA-seq analysis). Post-transcriptionally regulated gene lists, either up or down, were analyzed for miRNA target site enrichment using miRhub (Baran-Gale et al*.* 2013). In brief, miRhub scores a gene list based on the density of microRNA binding sites across the genes. A Monte-Carlo simulation is employed to score random gene lists of the same size (1000 permutations) and determine significance. The Limma package function RemoveBatchEffect() was used when applicable to correct for batch effect.

### Statistics

Significance was determined using a two-tailed unpaired Student’s *t* test unless explicitly stated otherwise. All correlations are reported with Pearson’s correlation coefficient.

## Results

### ***Exposure to iAs***^***III***^*** and MAs***^***III***^*** similarly impairs GSIS in INS1 832/13 cells***

**Fig. 1:**
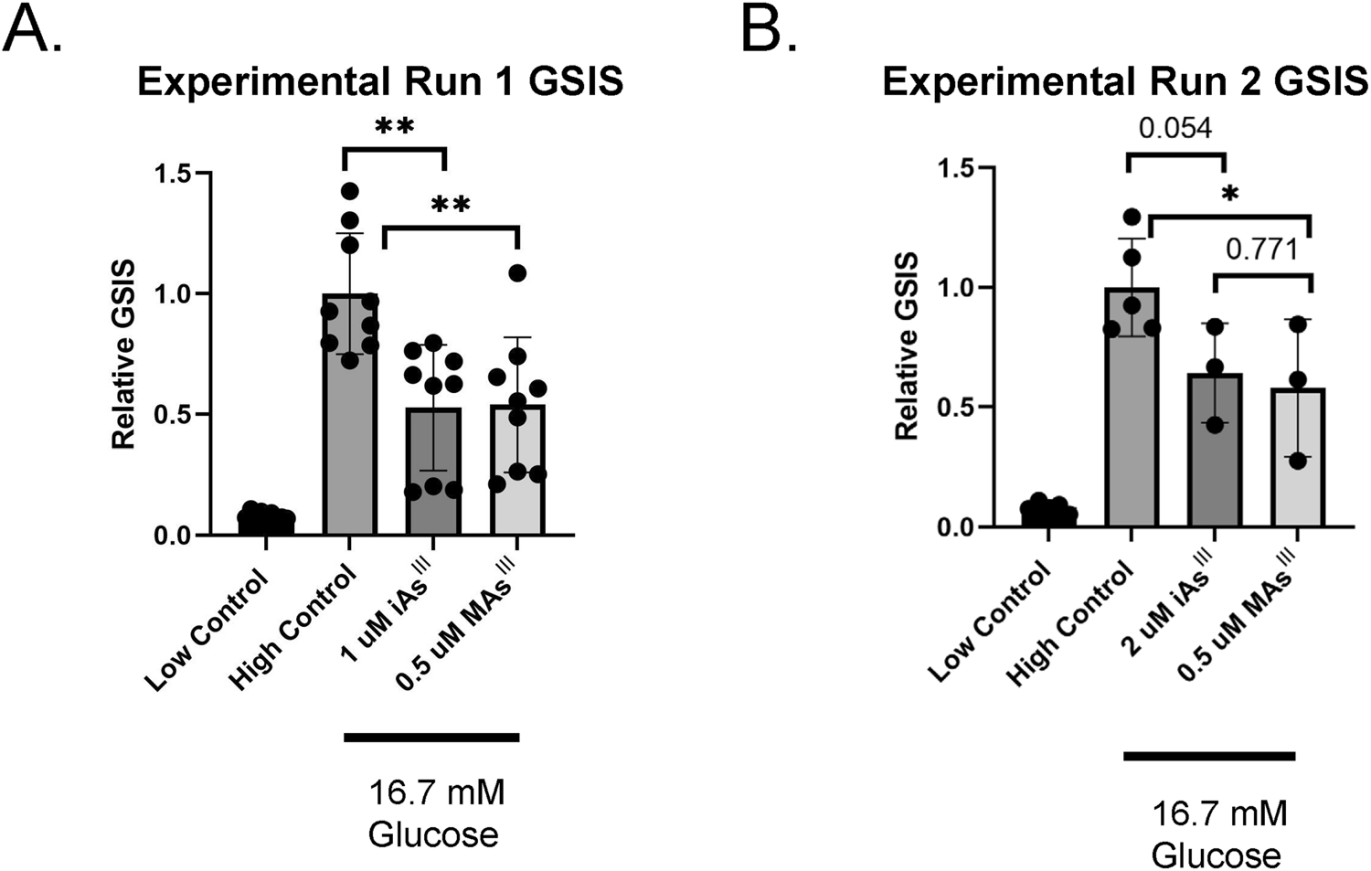
Exposure to arsenicals impairs glucose stimulated insulin secretion in INS-1 832/13 cells. INS-1 832/13 cells were exposed to 0.5 μM of MAs^III^, 1 μM of iAs^III^ (**A**), or 2 μM of iAs^III^ (**B**) for 24 h. Secreted insulin was quantified via insulin ELISA assays in technical and biological replicates of 3 and normalized to INS-1 832/13 cells incubated with high glucose and no arsenical. Experimental run 1 (**A**) and run 2 (**B**) were performed by two separate technicians with separately prepared arsenicals. Two-tailed unpaired Students *t* test was used to calculate the *p* values: *p value < 0.05, **p value < 0.01 arsenical treatment versus untreated high glucose control

We have previously shown that trivalent arsenicals, arsenite (iAs^III^) and its methylated metabolite MAs^III^, significantly impair GSIS in INS1 832/13 cells (Beck et al. 2019; Dover et al. 2018) and isolated murine islets (Douillet et al. 2013; Huang et al. 2019). In this study, we first sought to confirm that exposure to arsenicals leads to reduced GSIS in INS1 832/13 cells (Fig. 1). In the first experiment, we observed that 24-h exposure to either 1 μM iAs^III^ or 0.5 μM MAs^III^ significantly impairs GSIS (Fig. 1A). When repeated by a separate technician, with a new preparation of iAs^III^ and MAs^III^, we confirmed the effects, though the dose required for iAs^III^ (2 μM) was slightly higher (Fig. 1B). It is important to note that these concentrations have been shown previously to not be cytotoxic (Douillet et al. 2013; Dover et al. 2018; Huang et al. 2019).

### ***iAs***^***III***^*** and MAs***^***III***^*** alter miRNA profiles similarly in INS1 832/13 cells***

**Fig. 2:**
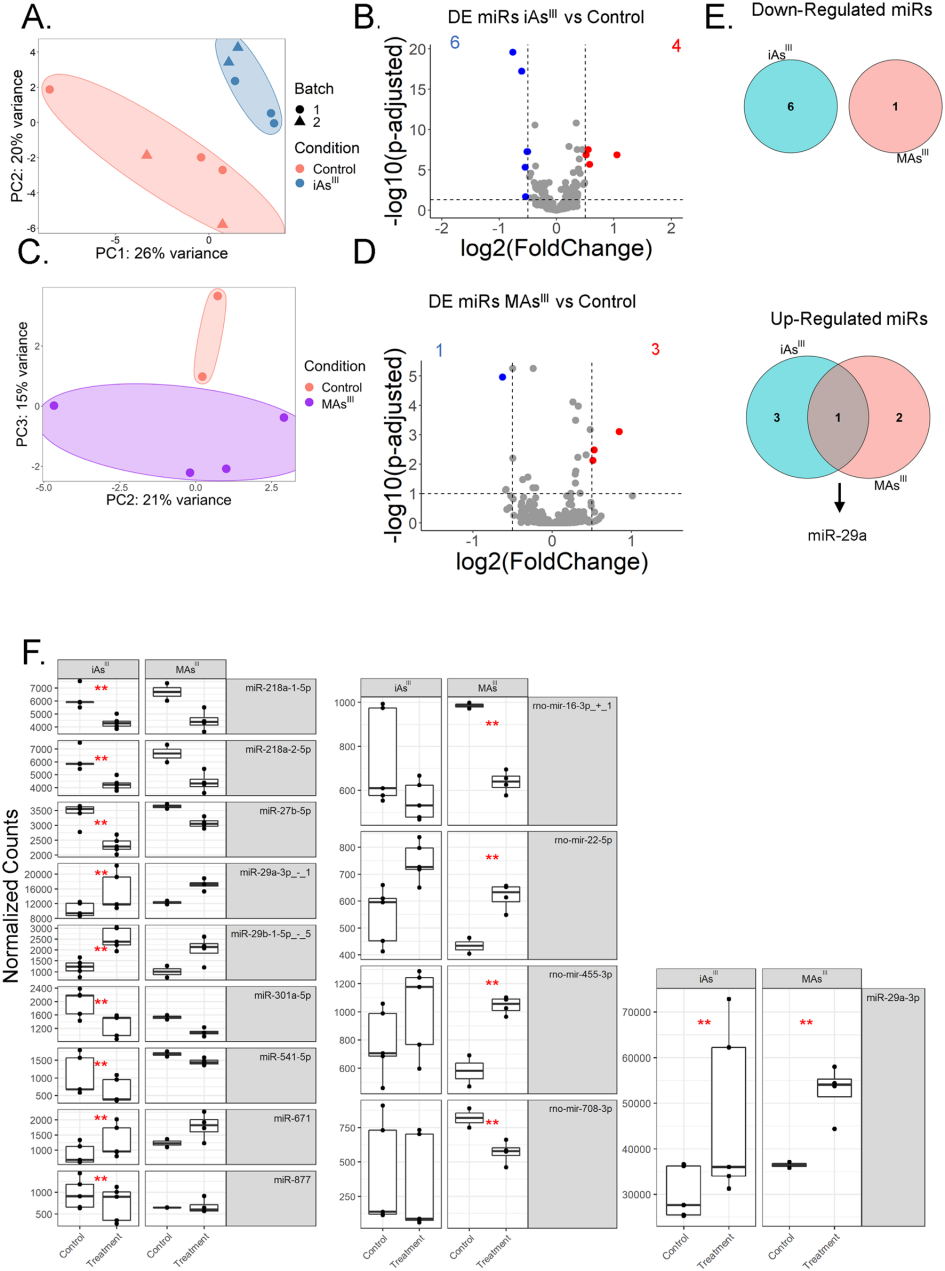
Exposure to iAs^III^ and MAs^III^ alters miRNA expression in INS-1 832/13 cells similarly. **A** Principal components analysis (PCA) plot of miRNA profiles in iAs^III^ treated cells compared to control group (untreated). Batch 1 and 2 were performed by separate technicians and therefore batch correction using the limma package was implemented. PCA plot was generated after variance stabilizing transformation (VST) of the data. **B** Volcano plot representing the differential expression (DE) analysis of miRNAs after iAs^III^ treatment. 10 significantly altered miRNAs are highlighted: 6 downregulated genes and 4 upregulated (*p* adjusted < 0.05, log2fold-change < − 0.5 or > 0.5, basemean > 500). **C** PCA plot of miRNA profiles in MAs^III^ treated cells compared to control group (untreated). PCA was generated after applying VST. All MAs^III^ treatments were performed by a single technician. **D** Volcano plot representing the DE analysis of miRNAs after MAs^III^ treatment. 4 significantly altered miRNAs are highlighted: 1 downregulated and 3 upregulated, in the MAs treatment compared to the control group (*p* adjusted < 0.05, log2fold-change < − 0.5 or > 0.5, basemean > 500). **E** Venn diagrams showing shared and uniquely altered miRNAs between iAs and MAs. **F** Significantly altered miRNA expression in iAs and MAs^III^ treatment groups. Normalized counts generated by DESeq2. The Benjamini–Hochberg method was used to calculate the adjusted *p* value: **p adjusted < 0.01

It is well established that miRNAs regulate insulin secretion (Kaur et al. 2020; Poy et al. 2004; Eliasson and Esguerra 2014; Latreille et al. 2014; Belgardt et al. 2015; Melkman-Zehavi et al. 2011) in beta cells and that perturbed miRNA expression leads to GSIS impairment (Bagge et al. 2012; Sun et al. 2019; Dooley et al. 2016; Pullen et al. 2011; Lovis et al. 2008). We have shown previously that iAs^III^ alters the miRNA profile of INS1 832/13 cells (Beck et al. 2019; Dover et al. 2018), yet it is not known whether MAs^III^ has a similar effect. INS1 832/13 cells were exposed to either arsenical for 24 h, after which we performed RNA isolation and small RNA-sequencing. Mapping statistics and read length distributions indicated high-integrity RNA and high-quality sequencing data (Supplemental Table 1 and Supplemental Fig. 1). We then analyzed these data using miRquant 2.0, a customized tool for miRNA quantification. Principal component analysis (PCA) of the miRNA profiles showed that iAs^III^-treated INS1 832/13 cells are distinctly separated from the untreated control group (Fig. 2A). In addition, we observed that the miRNA profiles between the two separate experiments are highly correlated (Supplemental Fig. 2A). Nonetheless, we applied batch correction for purposes of rigor and reproducibility. Differential expression (DE) analysis revealed that 10 miRNAs are significantly altered after exposure to iAs^III^: 4 upregulated and 6 downregulated (*p* adjusted < 0.05, log2fold-change < − 0.5 or > 0.5, basemean > 500) (Fig. 2B).

PCA of the miRNA profiles showed that cells exposed to MAs^III^ also clustered separately from the control group (Fig. 2C), though treatment was not responsible for the majority of variation between groups (Supplemental Fig. 3A, B). MAs^III^ treatment led to 4 significantly altered miRNAs: 3 upregulated and 1 downregulated (*p* adjusted < 0.05, log2fold-change < − 0.5 or > 0.5, basemean > 500) (Fig. 2D). Upon further analysis, we found no altered miRNAs that are shared between treatment groups in the downregulated group (miR-218-1, miR-218-2, and miR-301a) and one miRNA shared in the upregulated group (miR-29a) (Fig. 2E). Overall, iAs^III^ and MAs^III^ treated cells exhibit highly similar alterations in miRNA expression (Supplemental Fig. 4). That said, there are several altered miRNAs that are unique to each exposure, such as miR-877, which is altered only by iAs^III^, or miR-708, which is altered only by MAs^III^ (Fig. 2F).

### ***iAs***^***III***^*** or MAs***^***III***^*** exposure leads to unique changes in gene expression depending on the arsenical***

**Fig. 3:**
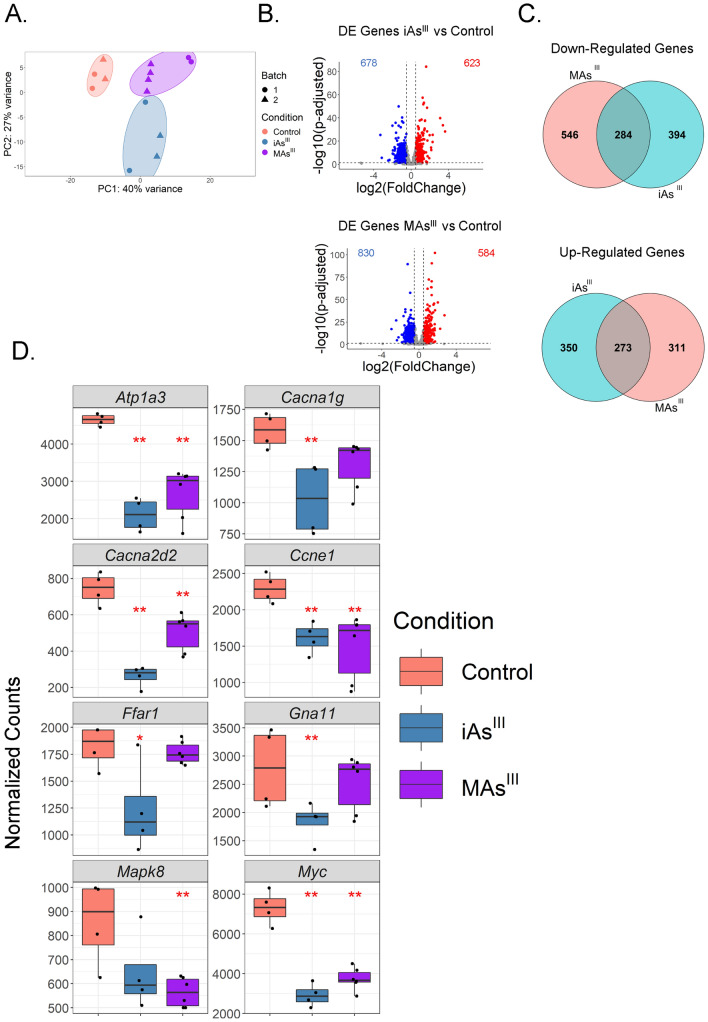
Exposure to iAs^III^ or MAs^III^ leads to unique changes in gene expression profiles in INS-1 832/13 cells. **A** PCA plot of gene expression profiles in iAs^III^ and MAs^III^ treated cells compared to the control group (untreated). Batch 1 and 2 were performed by separate technicians and therefore batch correction using the limma package was implemented. PCA plot was generated after applying VST. **B** Volcano plots representing the DE analysis of genes after iAs^III^ or MAs^III^ treatment (significance indicated by red or blue; *p* adjusted < 0.05, log2fold-change < − 0.5 or > 0.5, basemean > 500). **C** Venn diagrams showing shared and uniquely altered genes between iAs^III^ and MAs^III^. **D** Significantly altered gene expression in iAs^III^ and MAs^III^ treatment groups. Normalized counts generated from DESeq2 analysis. The Benjamini–Hochberg method was used to calculate the adjusted *p* value: **p adjusted < 0.01

To define the effects of arsenicals on gene expression in INS1 832/13 cells, we performed RNA-sequencing on samples exposed to either iAs^III^ or MAs^III^ for 24 h. PCA showed treatment-specific clustering (Fig. 3A). There was very little apparent batch effect for either treatment group (Supplemental Fig 2B); nonetheless, formal batch correction was applied to match the analysis of the miRNA data. DE analysis revealed significantly altered gene expression patterns in both iAs^III^ and MAs^III^ treated cells (*p* adjusted < 0.05, log2fold-change < − 0.5 or > 0.5, basemean > 500) (Fig. 3B). Specifically, we found 1251 genes significantly altered upon iAs^III^ treatment (623 up, 678 down) and 1414 after MAs^III^ treatment (584 up, 830 down) (Fig. 3B). Although we found that more genes are uniquely altered by each arsenical than are common to both treatments (Fig. 3C), it is evident that this is a consequence of the application of strict significance thresholds, because correlation analysis showed that iAs^III^ and MAs^III^ exhibit similar overall altered gene expression patterns (Supplemental Fig. 5). Analysis using the Enrichr tool (Kuleshov et al. 2016) showed that genes downregulated by iAs^III^ treatment are enriched in maturity onset diabetes of the young (MODY), T2D, insulin secretion, and calcium signaling pathways, as well as MAPK signaling and cell cycle (Supplemental Table 2). Notably, while the genes downregulated by MAs^III^ are also enriched in cell cycle and MAPK signaling, as well as glycolysis, they are not as over-represented in MODY, T2D, or calcium signaling pathways (Supplemental Table 3).

Among the genes, significantly downregulated after iAs^III^ treatment are *Atp1a3*, *Cacna1g*, *Ffar1,* and *Gna11* (Fig. 3D). These genes are involved in intracellular calcium signaling and insulin secretion (Supplemental Table 2). *Atp1a3* encodes a subunit isoform of a major Na + /K + ATPase pump (Salles et al. 2021), which is important for insulin secretion (Rorsman and Ashcroft 2018). *Cacna1g* codes for a component of the voltage-gated calcium channel, which is also essential for insulin secretion (Yang and Berggren 2006). Proper regulation of *Cacna1g* is imperative in beta cells, as reduced expression and activity leads to mitigated Ca2 + influx and weakened GSIS (Gilon et al. 2014). *Ffar1* encodes Fatty Acid Receptor 1, a G-coupled protein receptor (GPCR) known to promote insulin secretion in beta cells (Arora et al.; Kristinsson et al.; J. Liu et al.; Haber et al.). Finally, Gna11-mediated GPCR signaling is critical for autocrine potentiation of insulin secretion in mice (Sassmann et al. 2010).

Among the genes, most prominently downregulated after MAs^III^ treatment are *Mapk8*, *Ccne1*, and *Myc*, which are all involved in controlling beta cell survival and proliferation (Maedler et al. 2008; Varona-Santos et al. 2008; Cozar-Castellano et al. 2008; Karslioglu et al. 2011). Notably, specific genes encoding proteins that regulate insulin secretion, such as *Gna11* or *Ffar1*, which are suppressed after iAs^III^ treatment, are unaffected by MAs^III^. Overall, while there is high overall concordance in gene expression changes between iAs^III^ and MAs^III^, there are some altered genes and pathways that are unique to each arsenical.

### ***Exposure to iAs***^***III***^*** or MAs***^***III***^*** leads to unique changes in chromatin activity***

**Fig. 4:**
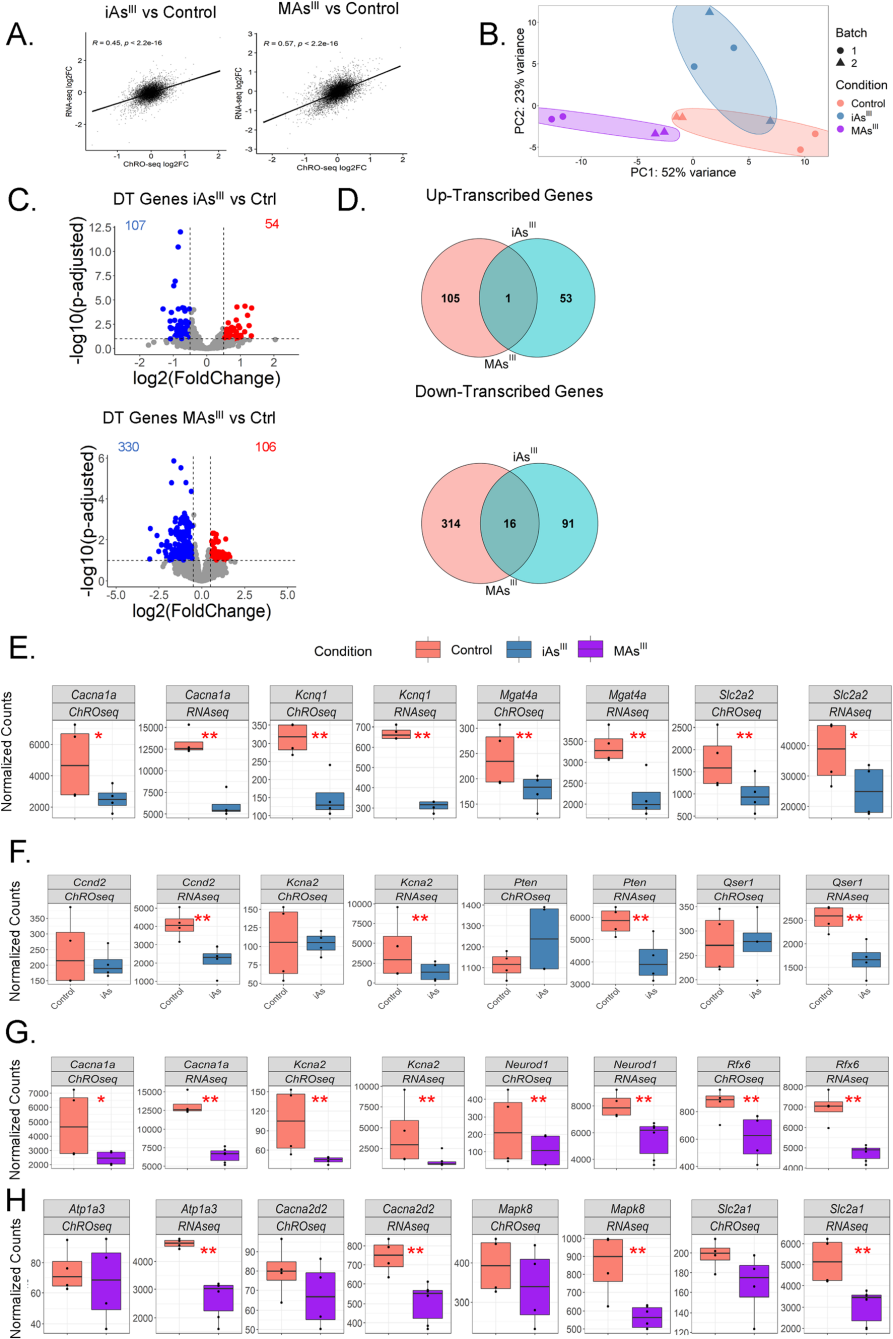
Exposure to iAs^III^ or MAs^III^ leads to unique changes in gene transcription profiles in INS-1 832/13 cells. **A** Correlation analysis between gene fold-changes at the RNA-seq level and ChRO-seq level (iAs^III^: R = 0.45, *p* value < 2.2e − 16; MAs^III^: R = 0.57, *p* value < 2.2e − 16). Only genes with baseMean > 500 in the RNA-seq data are shown. **B** PCA plot of altered gene transcription profiles in iAs^III^ and MAs^III^ treated cells compared to the control group (untreated). The limma package was used for batch correction between experimental runs. PCA plot was generated after applying VST. **C** Volcano plots representing differentially transcribed (DT) genes after iAs^III^ or MAs^III^ treatment (significance indicated by red or blue; *p* adjusted < 0.05, log2fold-change < − 0.5 or > 0.5). **D** Venn diagrams showing shared and uniquely altered genes between iAs^III^ and MAs^III^. **E** Significantly altered genes both transcriptionally and at the mRNA level in the iAs^III^ treatment group. **F** Significantly altered genes at only the mRNA level in the iAs^III^ treatment group. **G** Significantly altered genes both transcriptionally and at the mRNA level in the MAs^III^ treatment group. **H** Significantly altered genes at only the mRNA level in the MAs^III^ treatment group. Normalized counts generated from DESeq2 analysis. The Wald test was used to calculate *p* values: *p value < 0.05, **p value < 0.01

RNA-seq measures steady-state mRNA levels in the cell, whereas chromatin run-on sequencing (ChRO-seq) measures nascent transcription as well as promoter and enhancer activity landscapes (Chu et al. 2018). We performed ChRO-seq on samples exposed to either iAs^III^ or MAs^III^ for 24 h to define gene transcription profiles. Mapping statistics indicate high-quality sequencing data (Supplemental Table 4). We showed that changes in transcription (via ChRO-seq) after arsenical exposure are well-correlated with changes in steady-state expression (via RNA-seq) (Fig. 4A); however, as expected, they are not identical due in large part to post-transcriptional regulation (Blumberg et al. 2021). PCA showed that iAs^III^ and MAs^III^ confer distinct changes to transcriptional profiles (Fig. 4B). Both treatments led to significant changes in the transcription levels of hundreds of genes (*p* adjusted < 0.05, log2fold-change < − 0.5 or > 0.5) (Fig. 4C). Moreover, we found that the overlap in the changes between iAs^III^ and MAs^III^ is modest (Fig. 4D).

Genes coding for proteins involved in glucose transport, including *Slc2a2* (Glut2) (Rorsman and Ashcroft 2018; Thorens 2015; Thorens et al. 1990; Orci et al. 1990) and *Mgat4a* (López-Orduña et al. 2007; Ohtsubo et al. 2005), and calcium and potassium channels that promote insulin secretion, including *Cacna1a* and *Kcnq1*, are significantly downregulated by iAs^III^ at the levels of both transcription and steady-state expression (Fig. 4E). Other genes involved in beta cell survival (*Ccnd2* (Cozar-Castellano et al. 2008; Cozar-Castellano et al. 2006), *Pten* (Mziaut et al. 2020)) and insulin secretion (*Kcna2* (Tamarina et al. 2005), *Qser1* (Mahajan et al. 2018)) are suppressed by iAs^III^ only at the steady-state expression level but not at the transcriptional level (Fig. 4F), suggesting strong post-transcriptional regulation of those genes after iAs^III^ exposure. After MAs^III^ treatment, genes encoding two master transcription factors critical for the maintenance of beta cell function, *Neurod1* (Meulen and Huising 2015; Gu et al. 2010) and *Rfx6* (Smith et al. 2010; Piccand et al. 2014), are dramatically suppressed at the level of transcription (Fig. 4G), whereas several other genes (including genes coding for calcium channel Cacna2d2 and sodium pump Atp1a3a, both of which promote insulin secretion) are strong candidates for post-transcriptional regulation (Fig. 4H).

### *miR-29a is the top candidate master regulator of arsenic-induced post-transcriptional changes in gene expression*

**Fig. 5:**
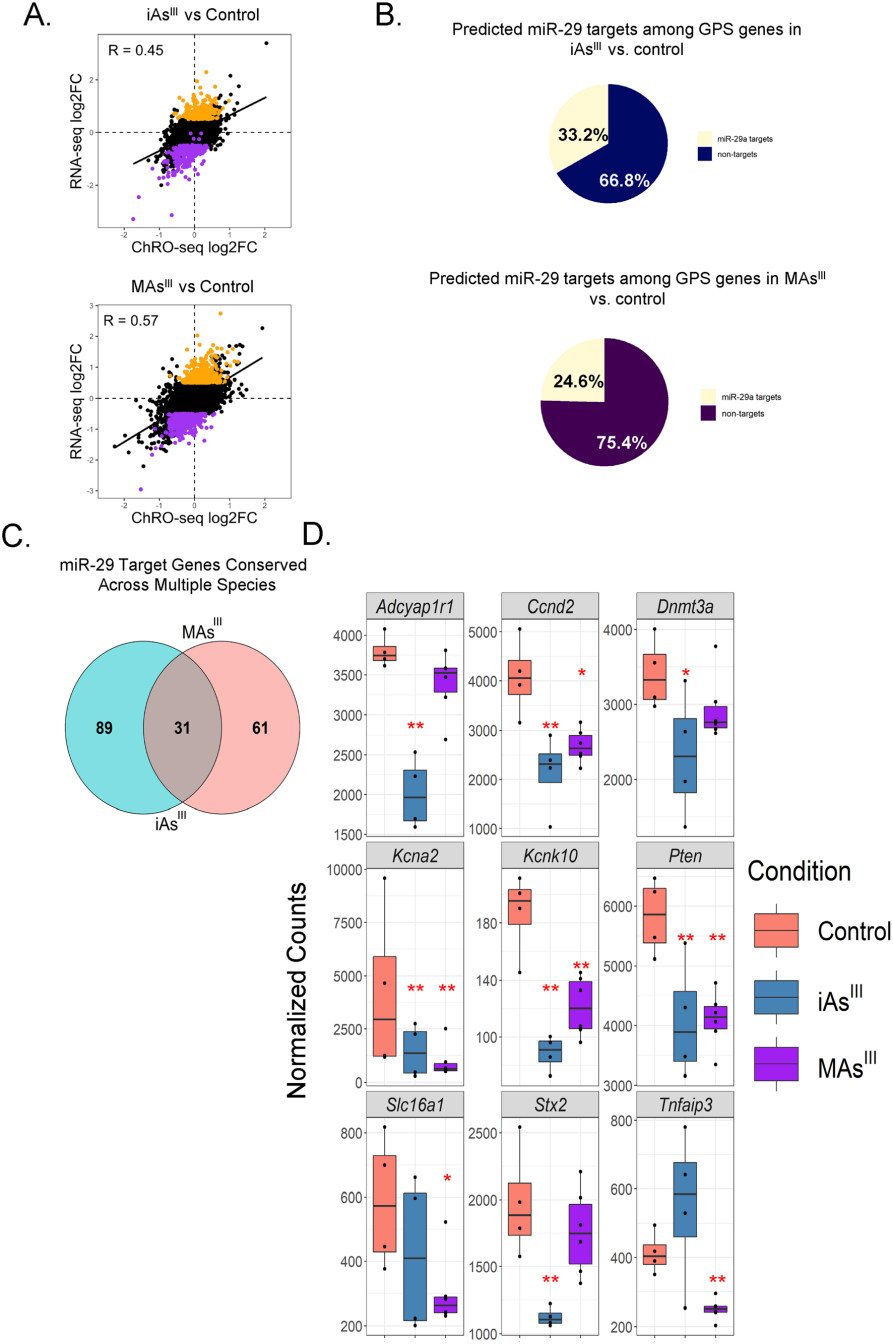
miR-29a is a candidate master regulator for arsenical-induced post-transcriptional changes in gene expression. **A** Two-factor analysis reveals genes significantly altered according to RNA-seq (*p* adjusted < 0.05, log2fold-change < − 0.5 or > 0.5) but not significantly altered according to ChRO-seq (*p* adjusted > 0.2) for iAs^III^ (top) and MAs^III^ (bottom). GPS genes are in purple and LPS genes are in orange. **B** Percentage of GPS genes that are miR-29a predicted targets in rats is shown. **C** Venn diagram representing overlap in conserved (between rat and two other species among human, mouse, and dog) miR-29a target GPS genes in iAs^III^ and MAs^III^ treatment conditions. **D** Significantly altered miR-29a target GPS genes in iAs^III^ only, MAs only, or both treatment groups. Normalized counts generated from DESeq2 analysis. The Benjamini–Hochberg method was used to calculate the adjusted *p* values: *p adjusted < 0.05, **p adjusted < 0.01

MiRNAs are prominent regulators of gene expression at the post-transcriptional level (Bartel 2004) and are well-known to confer robust control of beta cell function (Chakraborty et al. 2014; Kaur et al. 2020; Eliasson and Esguerra 2014). Therefore, we next sought to identify miRNAs that may be responsible for the regulation of genes controlled primarily at the post-transcriptional level after arsenical exposure. First, for each arsenical treatment condition, we performed DESeq2 two-factor integration analysis to identify genes solely post-transcriptionally regulated (PTR) (Methods, Fig. 5A). Among PTR genes, those that are increased at the mRNA level were subject to a loss of post-transcriptional suppression (LPS) and those that are decreased at the mRNA level were subject to a gain of post-transcriptional suppression (GPS).

To determine potential miRNA contributions to GPS genes, we next analyzed the GPS genes associated with either iAs^III^ or MAs^III^ treatment for enrichment of predicted targets of miRNAs that are significantly upregulated after exposure to the corresponding arsenical. We found that GPS genes associated with either arsenical are significantly enriched (*p* value for iAs^III^ and MAs^III^ = 0.000998) for predicted target sites of only one up-regulated miRNA, miR-29a. MiR-29a is a well-established regulator of insulin secretion and beta cell function (Bagge et al. 2012; Dooley et al. 2016; Pullen et al. 2011; Duan et al*.* 2019) and therefore an exciting candidate to investigate further. Approximately, 33% of the GPS genes after iAs^III^ exposure harbor predicted miR-29a target sites, and nearly 25% of the GPS genes after MAs^III^ exposure are also predicted to be targeted by miR-29a (Fig. 5B). Although there is a substantive number of miR-29a target GPS genes shared between MAs^III^ and iAs, there are also many that are unique to each arsenical (Fig. 5C). This finding suggests that although miR-29a is the top candidate master post-transcriptional regulator of gene expression in both iAs^III^ and MAs^III^ conditions, it may be involved in the control of overlapping but slightly different sets of genes depending on the arsenical.

We have highlighted some of the GPS genes, including two well-known miR-29a targets that are also important for beta cell health and identity, *Dnmt3a* (Hu et al. 2015; Dhawan et al. 2015) and *Pten* (Mziaut et al. 2020; Galimov et al. 2015) (Fig. 5D). In both treatment groups, we found genes important for insulin secretion (*Slc16a1*, *Stx2*, *Kcnk10*, *Kcna2*, and *Adcyap1r1*) (Pullen et al. 2011; Zhu et al. 2017; Kang et al. 2004; Liu et al. 2019) and beta cell survival (*Tnfaip3* and *Ccnd2*) (Ratajczak et al. 2021; Cozar-Castellano et al. 2006), though some of the genes are arsenical specific. In the MAs^III^ treatment group, we found *Slc16a1* and *Tnfaip3* to be significantly and uniquely downregulated. In the iAs^III^ treatment group, we found that *Adacyp1r1*, *Stx2*, and *Knca2* were significantly and uniquely downregulated. It is important to note that while *Kcna2* is significantly downregulated in both iAs^III^ and MAs^III^ groups (Fig. 5D), it is only a GPS gene in the iAs^III^ treatment group (Fig. 4F, G).

## Discussion

In this study, we used a multi-omics approach to identify miR-29a as a candidate master regulator of gene expression in INS-1 832/13 cells after exposure to either iAs^III^ or MAs^III^. The INS-1 832/13 cell line is a classical model for studying GSIS, as it maintains a robust response to glucose stimulus in culture (Hohmeier et al. 2000). It has been established that iAs^III^ and its metabolites are potent inhibitors of GSIS (Beck et al. 2019; Dover et al. 2018; Douillet et al. 2013; Huang et al. 2019), though the underlying mechanisms remain unclear. We sought to identify the effects of iAs^III^ and MAs^III^ on miRNA expression. Our experiments revealed miR-29a as a shared altered miRNA between arsenical treatments. MiR-29a is a well-established modulator of insulin secretion, in part through regulation of target genes such as syntaxin-1 (Bagge et al. 2012; Pullen et al. 2011; Duan et al*.* 2019; Filios and Shalev 2015; Baran-Gale et al*.* 2013; Bagge et al. 2012; Roggli et al. 2012).

We also identified key beta cell genes that are altered at the transcriptional and/or post-transcriptional levels, suggesting that multiple regulatory mechanisms may underlie arsenical-impaired GSIS. GSIS is a detailed process that utilizes numerous enzymes and channels within the beta cell. In brief, glucose enter through the glucose transporter Glut2 (gene name *Slc2a2*) and is metabolized to generate ATP. The increase in intracellular ATP closes the ATP-sensitive sodium/potassium channels, depolarizing the membrane and opening voltage-gated calcium channels. This results in a calcium influx, which ultimately leads to the secretion of insulin granules (Sabatini et al. 2019). After iAs^III^ treatment, we found that *Slc2a2* is significantly downregulated at the transcriptional level (Fig. 4E). Arsenicals have been hypothesized to use glucose permeases as a means to enter cells (Garbinski et al. 2019). Downregulation of *Slc2a2* may represent a protective mechanism against further arsenic damage or beta cells beginning to lose their identity and functionality. In MAs^III^ treated cells, we found that two genes encoding prominent factors of beta cell maintenance, *Neurod1* and *Rfx6*, are dramatically and uniquely suppressed. Neurod1 is a well-established transcription factor in the beta cell trajectory and is necessary for maintaining functional beta cell identity (Meulen and Huising 2015; Gu et al. 2010). Loss of the transcription factor Rfx6 is also associated with impaired glucose sensing and insulin secretion (Smith et al. 2010; Piccand et al. 2014). Loss of both or either *Neurod1* and *Rfx6* can result in an immature and non-functional beta cell.

We found that GPS (gain of post-transcriptional suppression) genes are significantly enriched for miR-29a targets in both iAs^III^ and MAs^III^ treatment groups (Fig. 5B). *Kcna2* is an example of a gene that is GPS after iAs^III^ treatment and is also a predicted miR-29a target. It encodes a protein that is a component of the voltage-gated potassium channel that is responsible for re-establishing the membrane potential of beta cells and thus preparing for the next wave of insulin granule secretion (Tamarina et al. 2005). Though *Kcna2* is not a GPS gene in the MAs^III^ treated cells, it is still significantly downregulated, highlighting that there may be miR-29a-independent mechanisms (at the transcriptional level) that are involved in *Kcna2* dysregulation under certain conditions. Interestingly, we found that a gene coding for another type of potassium channel, *Kcnk10*, is significantly downregulated in both iAs^III^ and MAs^III^ treated cells (Fig. 5D). *Kcnk10* is important for insulin secretion during metabolic stress (Kang et al. 2004) and its loss of expression may indicate that arsenicals increase susceptibility to diet-induced T2D. Previous studies have found that arsenic and high-fat diet, when administered together, work synergistically to impair insulin secretion in beta cells (Ahangarpour et al. 2018; Barrett 2011). Another gene of interest is *Adcyap1r1*, as it codes for a protein that potentiates insulin secretion and has been considered as a potential T2D therapeutic option (Filipsson et al. 1999; Inagaki et al. 1996; Liu et al. 2019; Marzagalli et al*.* 2015). Therefore, the loss of *Adycap1r1* is important to highlight in arsenic-induced T2D. Overall, our data reveal that, while there are some shared gene expression changes in iAs^III^ and MAs^III^ treated beta cells, iAs^III^ and MAs^III^ exert some unique effects on beta cell processes. Specifically, while miR-29a-mediated regulation of insulin secretion pathways appears to be prominent after iAs^III^ treatment, miR-29a-mediated regulation of beta cell survival and maintenance is likely to be of greater relevance in the context of MAs^III^ treatment.

iAs^III^ is an established diabetogen (Maull Elizabeth et al*.* 2012; Navas-Acien Ana et al. 2006), though the mechanisms by which it causes T2D have been poorly characterized. In isolated murine islets, it has been shown that arsenicals impair GSIS by potentially directly interfering with either the K_ATP_ or Cav1.2 channels and that GSIS could be restored to a degree using potassium channel blockers (Huang et al. 2019). Here, we find genes encoding components of both of these channels to be downregulated after arsenical exposure. Others have shown that arsenic induces apoptosis, indicating that impaired GSIS could be due to loss of beta cells (Pan et al. 2016; Fu et al. 2010). Furthermore, arsenicals have also been shown to change transcription and expression of insulin (Díaz-Villaseñor et al. 2006) and alter methylation patterns in cultured cells (Ehrlich et al. 2002), thus altering transcription patterns. Our findings support that arsenic does alter the transcriptional profiles in beta cells thereby leading to upregulation of miR-29a and miR-29a-mediated beta cell dysfunction. We also identified potential miR-29a-independent mechanisms, highlighting that arsenic-induced beta cell dysfunction is due to multiple disrupted processes. Future studies should be aimed at testing the hypothesis that inhibition of miR-29a can counteract the effects of arsenicals and at least partially restore GSIS in beta cells.

While we selected the INS-1 832/13 cell line for its robust GSIS response (Hohmeier et al. 2000), it does harbor limitations with regard to modeling inter-islet cellular communication. We do not know how arsenical exposure affects miRNA expression in other islet cell types and how this impacts GSIS from beta cells. Also, another important limitation is that the INS-1 832/13 cells are murine in origin. It will be important in the future to interrogate the effects of arsenicals on a newly established human beta cell line and/or human islets. While our bioinformatics pipeline does account for cross-species conservation, we cannot determine from the present study whether target genes identified in INS-1 832/13 cells are truly conserved in human beta cells without functionally validating in a human beta cell-like system.

Taken together, our data points to miR-29a as: (i) a top-candidate master regulator of gene expression in beta cells in response to both iAs^III^ and MAs^III^ treatment and (ii) a key regulator of genes involved in insulin secretion especially in the context of iAs treatment, and (iii) a key regulator of genes involved in beta cell maintenance and survival after either iAs^III^ or MAs^III^ treatment. Ultimately, our work suggests that miR-29a is a shared master regulator between iAs^III^ and MAs^III^ exposed beta cells, though it may act through the suppression of overlapping, but distinct sets of target genes.

## Supplementary Information

Below is the link to the electronic supplementary material.

Supplementary Supplemental Table 1: Mapping statistics generated by miRquant 2.0. Output statistics from miRquant 2.0 analysis. Supplemental Table 2: Top 30 enriched pathways for genes downregulated by iAsIII. Input genes were generated by DESeq2 analysis (RNA-seq: p adjusted < 0.05, log2fold-change < -0.5, basemean > 500) for iAsIII treated cells and the gene list was entered into Enrichr. Results were sorted by combined score and the top 30 were selected. Supplemental Table 3: Top 30 enriched pathways for genes downregulated by MAsIII. Input genes were generated by DESeq2 analysis (RNA-seq: p adjusted < 0.05, log2fold-change < -0.5, basemean > 500) for MAsIII treated cells and the gene list was entered into Enrichr. Results were sorted by combined score and the top 30 were selected. Supplemental Table 4: Mapping statistics for ChRO-seq. Output statistics from ChRO-seq analysis (XLSX 25 KB)

Supplementary Supplemental Fig. 1: Read length distribution for datasets generated by miRquant 2.0. Read length distribution of smRNA-seq data from INS-1 832/13 cells after 24-h exposure to 0.5 μM MAsIII, 1 μM of iAsIII (A), or2 μM of iAsIII (B) (TIF 115 KB)

Supplementary Supplemental Fig. 2: Correlation of miRNA and gene profiles across batches. A) Correlation analysis of miRNA profiles (basemean > 500 only) between different batches of iAs treatment experiments (R = 0.48, p value < 0.01). B) Correlation analysis of gene profiles (basemean > 500) between experimental runs of both iAsIII and MAsIII treatment. Experiments were done by different technicians (iAsIII: R = 0.92, p value < 0.01; MAsIII: R = 0.87, p value < 0.01) (TIF 1212 KB)

Supplementary Supplemental Fig. 3: Principal component analysis (PCA) with miRNA profiles generated by miRquant 2.0. PCA plots comparing miRNA profiles in MAsIII treated INS-1 932/13 cells with control cells. The plot was generated after VST transformation of the data. A) PC1 and PC2. B) PC1 and PC3 (TIFF 39 KB)

Supplementary Supplemental Fig. 4: Comparison of effects of iAsIII and MAsIII treatments on significantly altered miRNAs. Correlation of significantly altered miRNAs (p adjusted < 0.05, log2fold-change < -0.5 or > 0.5, basemean > 500) between iAsIII and MAsIII treatments (R = 0.97, p value < 0.01) (TIF 387 KB)

Supplementary Supplemental Fig. 5: Comparison of effects of iAsIII and MAsIII treatments on significantly altered genes. Correlation of significantly altered genes (p adjusted < 0.05, log2fold-change < -0.5 or > 0.5, basemean > 500) between iAsIII and MAsIII treatments (R = 0.85, p value < 0.01) (TIFF 329 KB)
