# Supplementary figures and images for: Candidate master microRNA regulator of arsenic-induced pancreatic beta cell impairment revealed by multi-omics analysis
Source: Arch Toxicol. 2022 Mar 21;96(6):1685–99. doi: 10.1007/s00204-022-03263-9 (PMC9095563; doi:10.1007/s00204-022-03263-9)

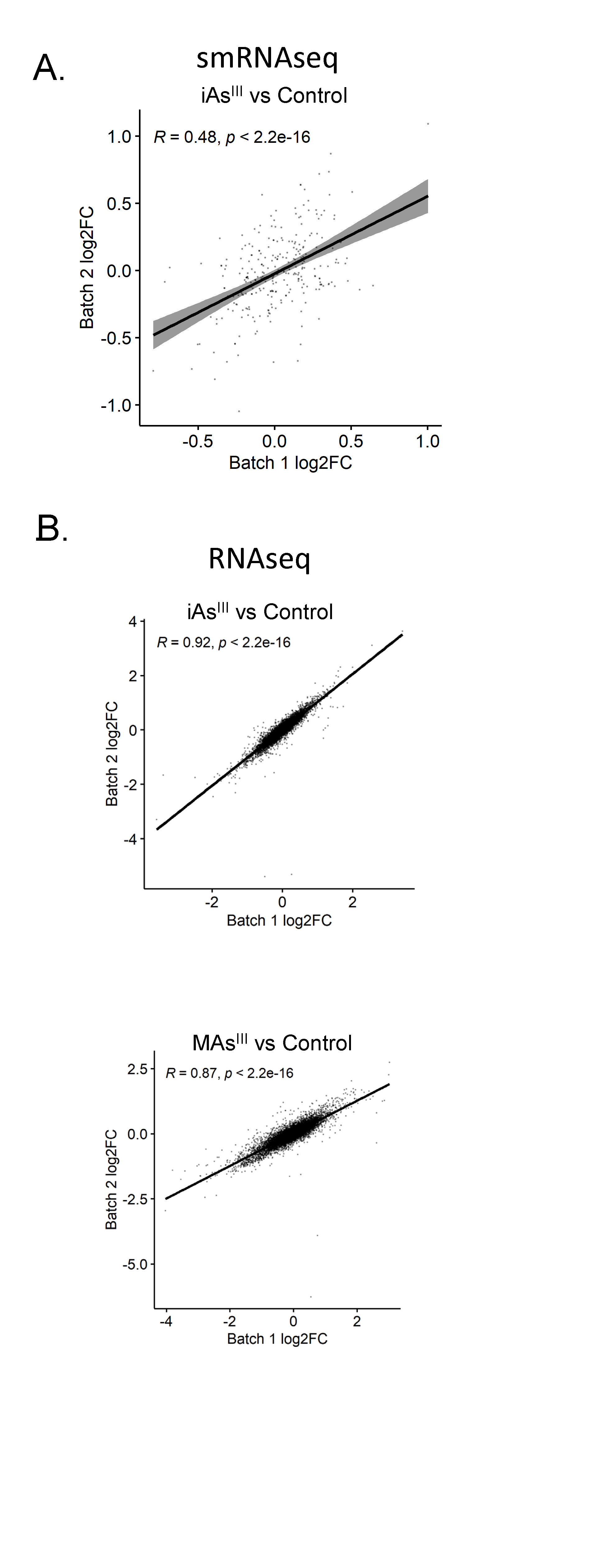

Supplement: Supplementary file 3 — Supplementary Supplemental Fig. 2: Correlation of miRNA and gene profiles across batches. A) Correlation analysis of miRNA profiles (basemean > 500 only) between different batches of iAs treatment experiments (R = 0.48, p value < 0.01). B) Correlation analysis of gene profiles (basemean > 500) between experimental runs of both iAsIII and MAsIII treatment. Experiments were done by different technicians (iAsIII: R = 0.92, p value < 0.01; MAsIII: R = 0.87, p value < 0.01) (TIF 1212 KB) [file 204_2022_3263_MOESM3_ESM.tif]

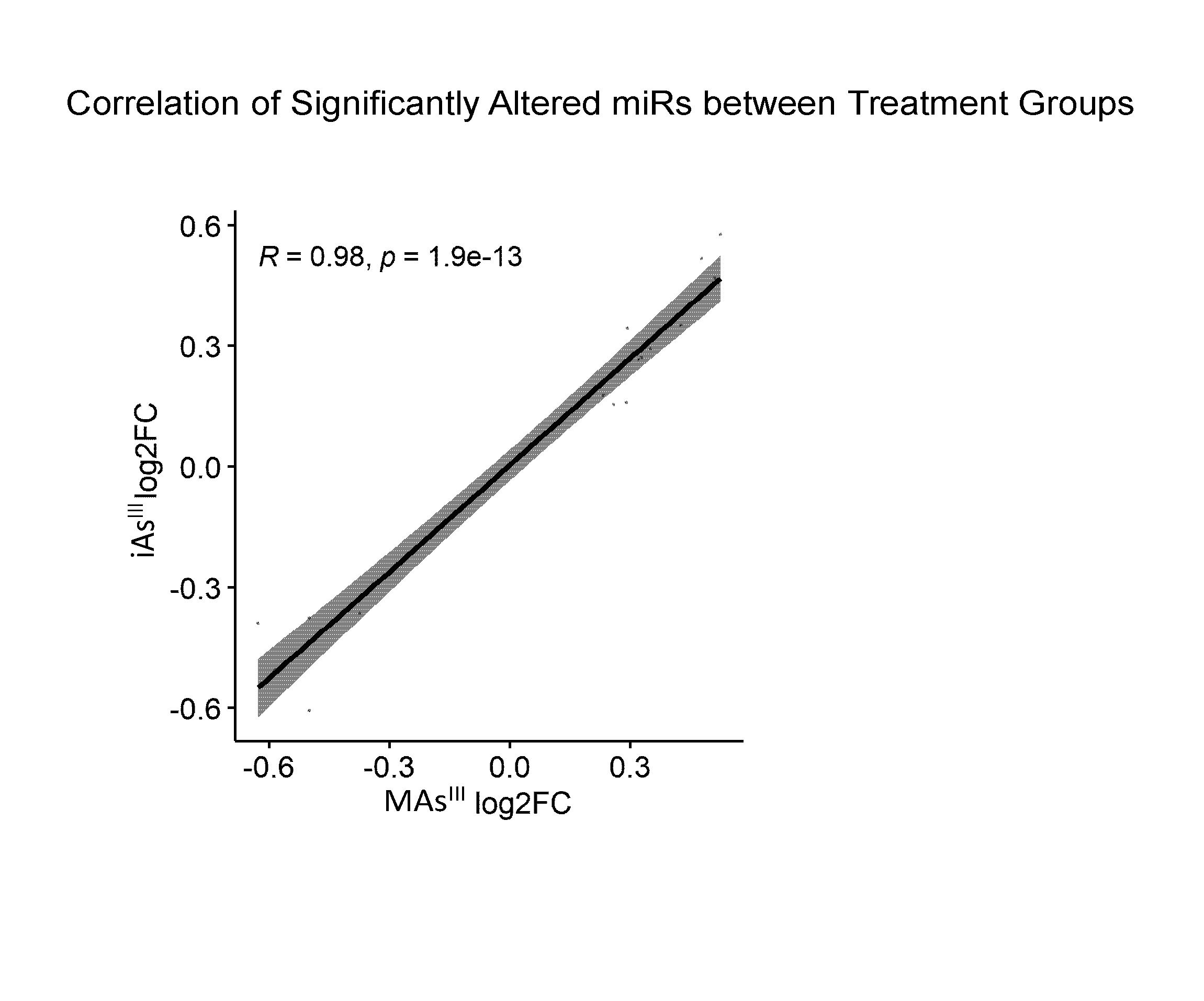

Supplement: Supplementary file 5 — Supplementary Supplemental Fig. 4: Comparison of effects of iAsIII and MAsIII treatments on significantly altered miRNAs. Correlation of significantly altered miRNAs (p adjusted < 0.05, log2fold-change < -0.5 or > 0.5, basemean > 500) between iAsIII and MAsIII treatments (R = 0.97, p value < 0.01) (TIF 387 KB) [file 204_2022_3263_MOESM5_ESM.tif]

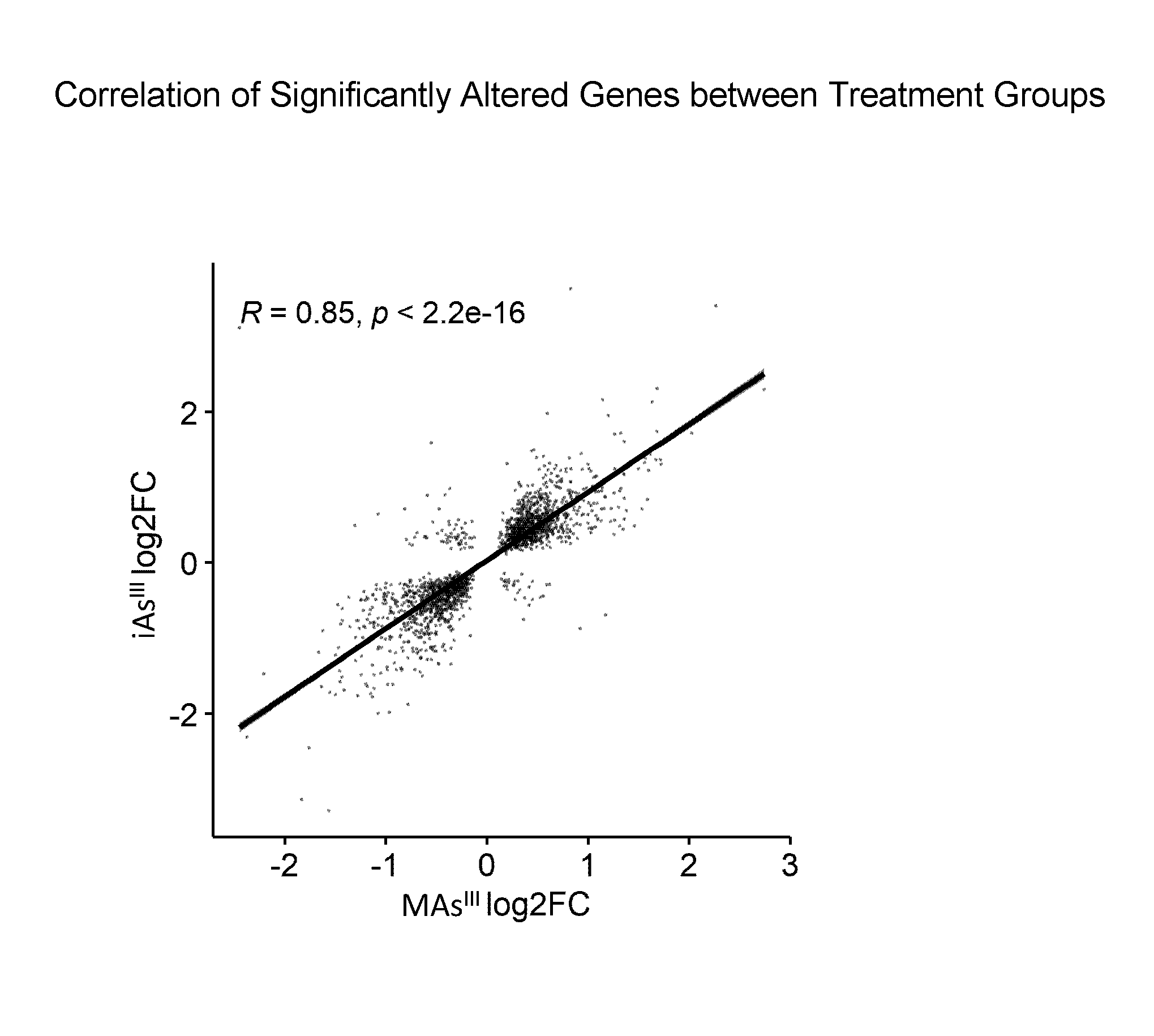

Supplement: Supplementary file 6 — Supplementary Supplemental Fig. 5: Comparison of effects of iAsIII and MAsIII treatments on significantly altered genes. Correlation of significantly altered genes (p adjusted < 0.05, log2fold-change < -0.5 or > 0.5, basemean > 500) between iAsIII and MAsIII treatments (R = 0.85, p value < 0.01) (TIFF 329 KB) [file 204_2022_3263_MOESM6_ESM.tiff]
